# Metabolic Epilepsies—Commemorative Issue in Honor of Professor Uwe Heinemann

**DOI:** 10.3390/ijms18112499

**Published:** 2017-11-22

**Authors:** Richard Kovács, Wolfram S. Kunz

**Affiliations:** 1 Institute for Neurophysiology, Charité—Universitätsmedizin Berlin, Corporate Member of Freie Universität Berlin, Humboldt-Universität zu Berlin, and Berlin Institute of Health, Berlin, Charitéplatz 1, 10117 Berlin, Germany; richard.kovacs@charite.de; 2 Department of Epileptology and Life & Brain Center, University of Bonn, Sigmund-Freud-Str. 25, D-53105 Bonn, Germany

Epilepsy is a very frequent, severe, and disabling neurological disorder with has a considerable disease burden worldwide. Although in about two thirds of patients, the epileptic seizures can be well controlled by available medications, novel treatment strategies for the remaining third of pharmaco-resistant patients are required, but the search for these strategies has been hampered by our poor understanding of the underlying pathology. Seizures are classically viewed as resulting from the imbalance of excitatory and inhibitory neuronal activities. Therefore, traditional epilepsy research has mainly focused on changes of synaptic transmission, caused by alterations in ion channels and neurotransmitter receptors. However, due to the recent dramatic progress in uncovering the genetic causes associated with epilepsy, there is an accumulating body of evidence indicating that seizure disorders should be considered in a much broader context. Especially, metabolic causes of seizures have recently obtained growing attention.

This special issue, “Metabolic Epilepsies”, covers a selection of recent research topics and current review articles about the contribution of alterations of brain metabolism to seizure activity. In particular, this special issue is dedicated to one of the most outstanding and influential scientists in the field of epilepsy research—Professor Uwe Heinemann (Figure 1)—who passed away on 8 September 2016 after a short, unforeseeable illness. The pioneering research work of Uwe Heinemann has broadened our understanding of epilepsy in many aspects of the pathological alterations of brain metabolism. The field of epilepsy research has lost a great personality and it will be difficult to fill the void he left behind. Those who had the luck of being part of the “Heinemann-lab” will miss his imagination, his immense lexical knowledge, and the playful ease with which he disentangled complex problems and relations.

For his studies on epilepsy, he was granted numerous prizes, such as the Michael-Prize for Epileptology, the Alfred Hauptmann prize, and the awards of American Epilepsy Society and the International League Against Epilepsy (ILAE). He was the president of the German Society for Epileptology and a member of many different scientific institutions and advisory boards, amongst others the Commission of ILAE and the European Academy of Epilepsy. In his role as the head of the Johannes Müller Institute for Neurophysiology at the Charité-Medical University-Berlin, the founding member of the Neuroscience Research Center, the Berlin School of Mind and Brain as well as the Excellence Cluster NeuroCure, he decisively shaped the reunified Berlin neuroscience scene. Nevertheless, if asked what he primarily took pride in, he always mentioned his doctoral students and postdocs first and foremost, many of whom have by now been granted permanent positions, or professorships and have become leading scientists in their respective fields.

It is incredibly difficult to summarize almost five decades of such a celebrated and productive scientific career, reflected in hundreds of publications, numerous inventions in the field of ion-sensitive electrodes, the brain slice technique, and epilepsy models in a short chapter.

His dissertation in Prof. Creutzfeldt’s lab in Munich dealt with the mechanism of EEG synchronization and EEG alterations in epileptic patients. Following his PhD (with a short stay at the University of Oxford (1971)), he joined Prof. Lux’ lab at the Max Planck Institute for Psychiatry in Munich (1971–1981), where he turned to the molecular aspects of epileptogenesis with a special interest in the alterations of ion homeostasis in the epileptic brain. This topic became his main research focus and accompanied him for the rest of his career, despite his interest and commitment to a multitude of areas including synaptic signaling and plasticity, and the organization of neuronal network activity, to name a few. After receiving a Heisenberg fellowship from the German Research Council (DFG) from 1981 to 1986, he first became a Professor of Physiology and Pathophysiology at Cologne University (1986–1993) and thereafter the chair of the Institute for Neurophysiology at the Charité (1993–2012). After 2012, he remained active as senior professor at the Neuroscience Research Center until his unexpected passing.

His initial studies on the alterations of ion-homeostasis in a multitude of in vivo and in vitro models of epilepsy branched off into a plethora of topics throughout the years. By investigating synaptic and non-synaptic ictogenesis, he also contributed to the general description of synaptic connectivity, plasticity- and frequency-dependent coupling of the entorhinal cortex-hippocampus-subiculum complex. The next step in this research area was the characterization of the generation of different types of network activity, such as gamma oscillations and sharp wave ripple complexes and their contribution to memory building and retrieval. 

Another path led to the discovery of disturbances in neurovascular coupling and the function of the blood–brain barrier during recurrent seizures. These events were found to initiate the transformation of astrocytes leading to impaired spatial buffering of potassium and increased seizure susceptibility. Studying alterations in chronic epileptic tissue from patients undergoing epilepsy surgery and in animal models of epilepsy, he suggested and tested several mechanisms behind pharmaco-resistance, (i.e., the loss of responsiveness to several AED regimens). As the main energy consumer of the brain is the sodium potassium ATPase, accounting for ~50% of the ATP usage even during physiological network activity, his findings on seizure-associated alterations of the ion homeostasis paved the way for the investigation of the energetic aspects of epileptic activity. Thus, he was amongst the first to describe the seizure-dependent formation of reactive oxygen and nitrogen species and their contribution to mitochondrial dysfunction and subsequent neuronal injury. 

Two of Uwe Heinemann’s latest studies are printed in this special issue, presented by his co-workers. The study by Schoknecht et al. [1] aims to describe the metabolic consequences (oxygen consumption rate and mitochondrial activation) of different forms of ictal and interictal activity by using a combination of experimental and metabolic modeling approaches. The elegance of this study lies in the combined use of the γ-aminobutyric acid (GABA) receptor antagonist bicuculline and the M-current inhibitor XE-991, which allows for arbitrary switching between the different activity forms during recording. The paper by Angamo et al. [2] is a follow-up of a previous study of Uwe Heinemann, in which he described the contribution of intrinsic lactate as an energy substrate to the maintenance of synaptic signaling and ion-homeostasis. In chronic epileptic tissue, however, the expression pattern of monocarboxylate transporters (the main route of lactate transport) is altered, implicating disturbances in lactate metabolism. The present study describes an anti-seizure effect of lactate uptake inhibitors mediated by substrate deprivation and subsequent activation of adenosine receptors.

In addition to the two latest studies of Uwe Heinemann, 9 further papers consisting of 7 reviews and 2 research articles have been included in this issue as detailed in Table 1. The topics covered include epilepsy-associated metabolic alterations, metabolic diseases associated with epilepsy, and metabolic effects of antiepileptic medications. 

The review of Bazzigaluppi et al. [3] provides a summary of clinical and preclinical observations showing the metabolic dysregulation in epileptogenesis, but also during seizure initiation and termination, and even in the inter-ictal period. The research paper of Lai et al. [4] focuses on one potential mechanism of hippocampal neuronal damage during status epilepticus. Several reviews [5,6,7] included in this special issue give an overview of the large number of very heterogeneous metabolic diseases associated with epilepsy. These include mitochondrial diseases (the review of Hikmat et al. [5] is focused on epilepsy-associated disorders due to pathogenic mutation in the mitochondrial DNA polymerase γ), a large number of epilepsy-associated disorders related to inborn errors of metabolism (reviewed by Sharma & Prasad in [6]), and Lafora disease (reviewed by Sullivan et al. in [7]), a devastating polyglucosan storage disorder. Two further reviews by Kovac et al. [8] and Pearson-Smith & Patel [9] give an overview about the metabolic changes associated with epilepsy highlighting the different views on the origin and the role of reactive oxygen species in acquired epilepsy. 

Finally, two further papers describe the metabolic effects of antiepileptic medications. The research paper by Kudin et al. [10] is focused on the reasons for the devastating side effects of valproic acid in epilepsy patients with an underlying mitochondrial disease and the review by Fan et al. [11] discusses the effects of anti-epileptic drugs on growth and bone metabolism.

In conclusion, we would like to thank the authors who contributed to this issue—since many of them were good friends and former pupils of Uwe Heinemann, we are in good hope that his legacy will be carried on.

## Figures and Tables

**Figure 1:**
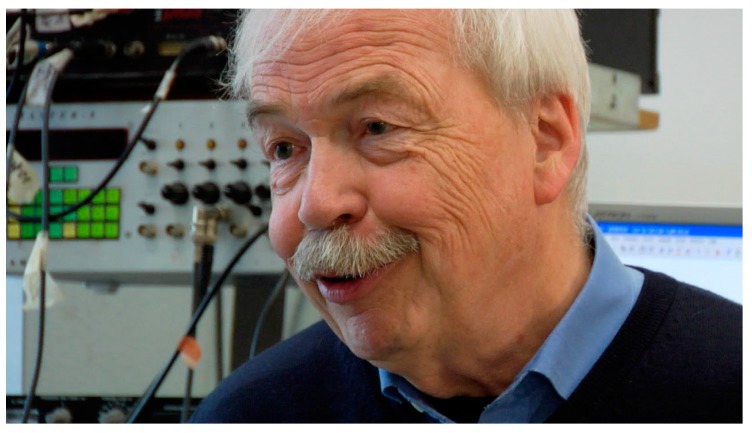
Uwe Heinemann (born 17 February 1944 in Genthin; died 8 September 2016 in Berlin).

**Table 1:** Overview of articles published in this special issue entitled “Metabolic Epilepsies”.

Authors	Title	Topic	Publication Type
Schoknecht et al. [1]	Event-Associated Oxygen Consumption Rate Increases ca. Five-Fold When Interictal Activity Transforms into Seizure-Like Events In Vitro	Epilepsy-associated metabolic alterations	research article
Angamo et al. [2]	Contribution of Intrinsic Lactate to Maintenance of Seizure Activity in Neocortical Slices from Patients with Temporal Lobe Epilepsy and in Rat Entorhinal Cortex	Epilepsy-associated metabolic alterations	research article
Bazzigaluppi et al. [3]	Hungry neurons: metabolic insights on seizure dynamics	Epilepsy-associated metabolic alterations	review
Lai et al. [4]	Mitochondrial Dysfunction Mediated by Poly(ADP-Ribose) Polymerase-1 Activation Contributes to Hippocampal Neuronal Damage Following Status Epilepticus	Epilepsy-associated mitochondrial dysfunction	research article
Hikmat et al. [5]	Understanding the Epilepsy in POLG Related Disease	Mitochondrial disease associated with epilepsy	review
Sharma & Prasad [6]	Inborn Errors of Metabolism and Epilepsy: Current Understanding, Diagnosis, and Treatment Approaches	Metabolic diseases associated with epilepsy	review
Sullivan et al. [7]	Pathogenesis of Lafora Disease: Transition of Soluble Glycogen to Insoluble Polyglucosan	Metabolic disease associated with epilepsy	review
Kovac et al. [8]	Metabolic and Homeostatic Changes in Seizures and Acquired Epilepsy—Mitochondria, Calcium Dynamics and Reactive Oxygen Species	Metabolic changes associated with acquired epilepsy	review
Pearson-Smith & Patel [9]	Metabolic dysfunction and oxidative stress in Epilepsy	Metabolic changes associated with acquired epilepsy	review
Kudin et al. [10]	Mitochondrial Liver Toxicity of Valproic Acid and Its Acid Derivatives Is Related to Inhibition of α-Lipoamide Dehydrogenase	Metabolic effects of antiepileptic medications	research article
Fan et al. [11]	The Impact of Anti-Epileptic Drugs on Growth and Bone Metabolism	Metabolic effects of antiepileptic medications	review

## Abbreviations

POLG	DNA polymerase γ
ILAE	International League Against Epilepsy
